# Genome-wide systematic characterization of PHT gene family and its member involved in phosphate uptake in *Orychophragmus violaceus*

**DOI:** 10.1186/s12864-025-12091-x

**Published:** 2025-09-29

**Authors:** Jianjian Liu, Shuangshuang Wang, Tianyi Ying, Changming Zou, Jianrong Zhao

**Affiliations:** 1 College of Resource and Environment, Anhui Science and Technology University, Fengyang, China; 2 State Key Laboratory of Tropical Crop Breeding, Institute of Tropical Bioscience and Biotechnology, Sanya Research Institute, Chinese Academy of Tropical Agricultural Sciences, Sanya, China

**Keywords:** *Orychophragmus violaceus*, Phosphate, PHT transporter, Gene duplication, Expression pattern

## Abstract

**Background:**

Phosphorus (P) is an indispensable mineral element which plays crucial roles in plant development and production. While it is widely acknowledged that phosphate transporters (PHT) facilitate the absorption of P, detailed identification and characterization of the PHT genes in *Orychophragmus violaceus* remains unexplored.

**Results:**

Here, a total of 22 *OvPHT* genes were identified from high-quality genome of *O. violaceus* and could be categorized into four distinct subfamilies, based on the phylogenetic analysis. Synteny analysis revealed that the *OvPHT* gene family underwent nine segmental duplication events and two tandem duplication events. Additionally, a detailed analysis of the Ka/Ks ratios indicated that these duplicated gene pairs primarily experienced purifying selection. Expression patterns of these *OvPHT* genes across various tissues, including leaves, roots, stems, and flowers, showed differential but partial-overlapping patterns. *OvPHT1* members were predominantly expressed in roots and induced by P starvation. Conversely, *OvPHT4* members showed high expression levels in all tested tissues, except for roots; the expression levels of *OvPHT2* and *OvPHT3* members were mainly detected in leaves and flowers, respectively. Moreover, OvPHT1;1 was localized in the plasma membrane. Overexpression of *OvPHT1;1* in rice could enhance P uptake under low-P condition.

**Conclusion:**

In summary, this systematic analysis provides detailed information for a better understanding of *OvPHT* genes and identifies candidate genes for further exploration of P-efficient utilization in *O. violaceus*.

**Supplementary Information:**

The online version contains supplementary material available at 10.1186/s12864-025-12091-x.

## Introduction

Phosphorus (P) is one of the essential macronutrients involved in plant growth and development, such as nucleic acid synthesis, signal transduction processes, and serving as a key component in ATP and phospholipids [1, 2]. Nitrogen (N), P, and potassium (K) fertilizer plays a predominant role in modern agricultural production. While N can be replenished via synthetic ammonia production and biological fixation by nodule bacteria, and K is abundant in minerals and seawater, P lacks such renewable pathways, making it a finite resource [3]. Approximately 70% of the global cultivated land soils are plagued by P deficiency, significantly hindering crop yields and quality [4–6]. As the main form of P that is acquired by plant roots, inorganic phosphate is unevenly distributed and easily chemical fixed by aluminum and iron in the soil [7]. Therefore, a large amount of P fertilizers has been applied to maintain high crop yields in agricultural ecosystems. Enhancing P utilization efficiency is imperative not only for ensuring food production but also urgently required to safeguard phosphorus fertilizer resources.

Plants have developed various mechanisms to cope with low-P stress during the process of evolution [8, 9]. Organic acids and phosphatases, such as malate and citrate, secreted by plant roots, can liberate P from immobilized-P, thereby boosting its availability in the rhizosphere soil [10, 11]. Meanwhile, modifications in the architecture of the plant root system are triggered to adapt to varying levels of Pi availability. For example, the growth of plant primary root was strongly repressed by low-Pi stress, while the increase of number of the lateral roots and root hairs could enhance the plant's ability to efficiently acquire P from the soil [11, 12]. Over 80% land plants could form mutualistic symbioses with arbuscular mycorrhizal fungi (AMF). As ancient mutualistic associations in terrestrial ecosystems, AM symbiosis plays an important role in enhancing mineral nutrient supply. In particular, AMF could provide a mycorrhizal pathway to enhance the plant’s P uptake ability[13]. Furthermore, the activation of high-affinity phosphate transporter (PHT) genes is also crucial. Phosphate starvation-induced PHT genes play a dominant role in plant Pi supply under low-P condition [9, 14, 15].

The P concentration in the plant cytoplasmic is commonly much higher than that in the soil solution [16]. This suggests the plants need to rely on specialized transport systems to acquire phosphate. It has been reported that the transmembrane transport of phosphate can be mediated by plasma membrane-localized phosphate transporter proteins with different affinities. With the development of whole-genome sequencing, PHT family genes have been identified in numerous plants, such as Arabidopsis, rice, tomato (*Solanum lycopersicum*), soybean (*Glycine max*), *Cucumis melo L.* and maize (*Zea mays*) [17–21]. Among these, phosphate transporter-1 (PHT1) genes have been most widely demonstrated to have irreplaceable roles in the acquisition and translocation of Pi in plants [22]. To date, the biological properties and physiological functions of most OsPHT1 transporters have been characterized in rice (*Oryza sativa*). For instance, OsPHT1;1, OsPHT1;2 and OsPHT1;8 are involved in not only phosphate uptake but also phosphate root-to-shoot translocation [18, 23]. OsPHT1;3 is significantly induced by phosphate starvation, and mediates Pi homeostasis under extremely low Pi concentration conditions (below 5 μM) [24]. Unlike root-expressed PHTs, OsPHT1;7 has been demonstrated to mediate Pi accumulation in anthers and play a crucial role in Pi recycling [25]. Arabidopsis AtPHT2;1, *Triticum aestivum* TaPHT2;1 and *Oryza sativa* OsPHT2;1 are the chloroplast-envelope-localized Pi transporter. Under P sufficiency, a significant decrease in P accumulation was observed in the *atpht2;1* and the TaPHT2;1 knock-down lines, but not in the *ospht2;1* [26–28]. The mitochondrial-localized Pi transporter PHT3;1 plays an indispensable role in respiration and normal growth for *Arabidopsis* [29]. The PHT4 family consists of six members (AtPHT4;1–4;6) in *Arabidopsis*, which play diverse roles in plant growth and development, including maintaining phosphate homeostasis in various subcellular organelles, defending against pathogens, and regulating carbon metabolism [30, 31]. These specialized transporter proteins, with their unique functions and expression patterns, allow plants to efficiently acquire and distribute P, ensuring optimal growth and development even under varying soil P-conditions.

*O. violaceus*, commonly known as "eryuelan" due to its blooming in February of the lunar calendar, is widely cultivated in northern China. As an ornamental species of Brassicaceae, its seeds are rich in unsaturated fatty acids, particularly dihydroxy fatty acids, making it a valuable oil economic crop [32–34]. P plays a crucial role in the oil synthesis process of oilseed crops, particularly in Brassicaceae species such as *Brassica napus* and *O. violaceus*, P deficiency severely restricts the synthesis and metabolism of fatty acids and phospholipids in plants [35, 36]. As a green manure, *O. violaceus* possesses the capability to release available nutrients and improve rhizosphere soil microecology [34, 37–39]. Recently, with the release of the high-quality *O. violaceus* genomic, our understanding of the molecular mechanisms underlying Pi-uptake in these plants has significantly enhanced, accelerating the breeding process [40, 41]. Here, a total of 22 PHT genes were identified from the genomic dataset of *O. violaceus*. We analyzed their protein physicochemical properties, evolution history, gene structure, conserved motifs, collinearity, and tissue-specific expression profiles. The expression patterns of OvPHTs in response to different Pi treatments were examined. Additionally, the subcellular localization and functional characterization of OvPHT1;1 in P-deficiency response were investigated. Taken together, these results will lay a solid foundation for further investigatation the role of the *OvPHT* gene family in response to low-P stress in *O. violaceus*.

## Result

### Identification and characterization of OvPHTs in O. violaceus

A total of 22 OvPHT genes were renamed based on the homology with AtPTs. The detailed physicochemical properties of the OvPHT proteins were analyzed, including sequence IDs, protein characteristics and chromosomal locations (Table S1). The amino acid lengths and molecular Weights of the 22 OvPHT proteins ranged from 351 (OvPH3;1) to 584 (OvPHT2;1) and from 36.43 kDa to 63.61 kDa, respectively. Additionally, the isoelectric points ranged from 8.81 to 9.53. The GRAVY index analysis revealed that all the OvPHT proteins were hydrophilic. The information of chromosomal location showed 22 *OvPHTs* were randomly distributed on nine chromosomes in *O. violaceus*. In addition, the sub-cellular localization of OvPHTs were predicted using the WoLF PSORT online website. Overall, the subcellular localization of OvPHTs was comparable to those observed for PHTs in other plant species. OvPHT1 and OvPHT4 subfamily members were localized in the plasma membrane. Whereas OvPHT2s and OvPHT3s were localized in the chloroplasts and mitochondria, respectively.

### Phylogenetic analysis of PHT genes in O. violaceus

**Fig. 1:**
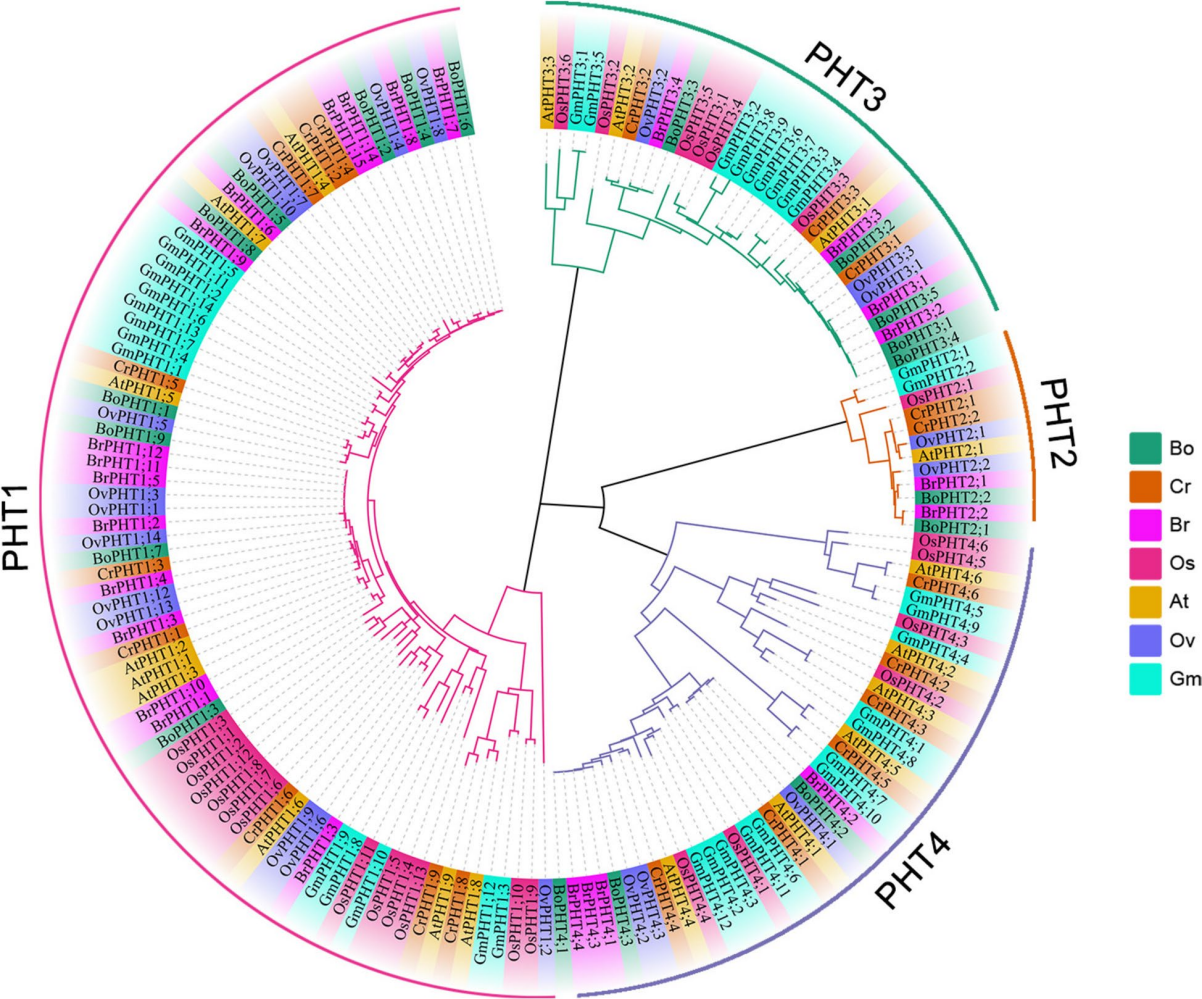
Phylogenetic analysis of PHTs proteins from *A. thaliana*, *O. sativa*, *B. rapa, C. rubella, and B. oleracea, G. max* and *O. violaceus*. The different colors branches and leaves represent four different PHT subfamilies, and plant species, respectively

As shown in Fig. 1, the 22 OvPHTs can be categorized into four subfamilies (PHT1, PHT2, PHT3, PHT4). As the largest group, the OvPHT1 subfamily contains 14 members, and all OvPHT1 genes exhibit high homology with the PHT1 genes in Arabidopsis, with the exception of OvPHT1;11 and OvPHT1;12. PHT2 subfamily was the smallest group and comprising only two OvPHT2 members. Both the PHT3 and PHT4 subfamilies contain three members each.

**Fig. 2:**
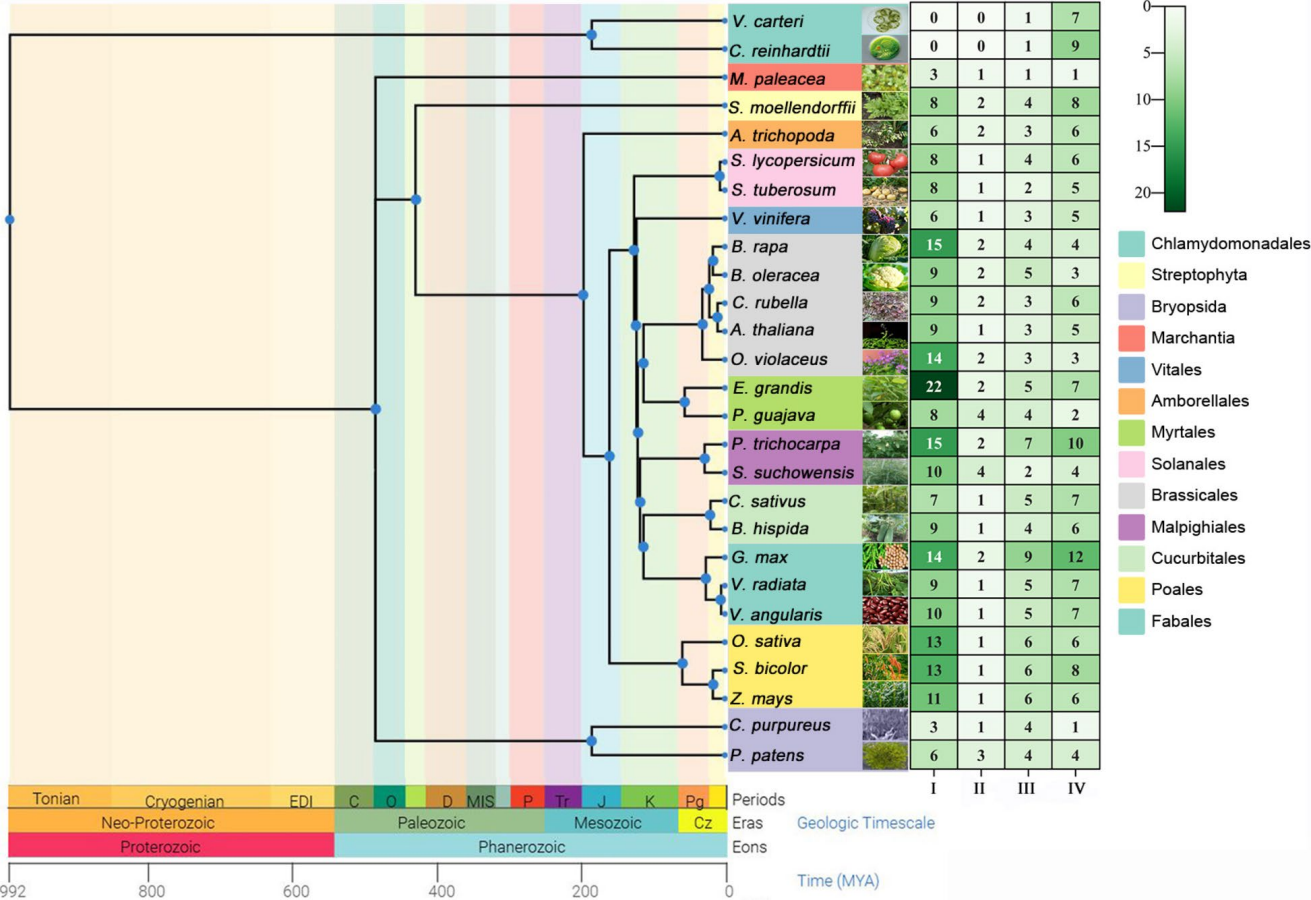
Phylogeny and diversity of PHT gene in 25 species

To further uncover the evolutionary history of the PHT gene family, we selected 28 species to construct a time tree and list the numbers of PHTs in each subfamily of these species (Fig. 2). No members of the PHT1 and PHT2 subfamily were found in *Volvox carteri* and *Chlamydomonas reinhardtii*, while PHT4 subfamily contain the majority of PHT genes. The number of *PHT1* family gene members in the Vitales, Amborellales, Solanales and Cucurbitales ranges between 6 and 9, whereas significantly higher numbers, ranging between 9 and 14, are found in Polales and Fabales. A similar evolutionary distribution pattern is also evident in the *PHT3* gene family. Interestingly, the number of *PHT2* subfamily members remained highly conserved among diverse land plants, hinting at a limited degree of functional diversification within this subfamily. Additionally, the expansion pattern of the PHT gene was inconsistent with different plant families. For instance, the number of PHT1 and PHT4 genes in *Eucalyptus grandis* had three times compared with those in *Psidium guajava*. But in Brassicales species, the expansion of PHT1 subfamily plays a major role in the difference of total gene numbers between *Arabidopsis thaliana* and *O. violaceus*.

### Gene structure, conserved protein domain and motif analyses of OvPHTs

All PHT1 genes contain the phosphate-H^+^ symporter domain (pfam00083). PHT3 subfamily members contain two mitochondrial carrier domains (pfam00153). Besides, PHT2 and PHT4 have a phosphate/sulfate permease domain (COG0306) and major facilitator superfamily domain (cd17380), respectively. These distinct conserved domains within each subfamily of *OvPHT* genes in *O. violaceus* likely underlie their unique responses to phosphorus-deficiency stress, highlighting the diverse roles these genes play in P homeostasis.

**Fig. 3:**
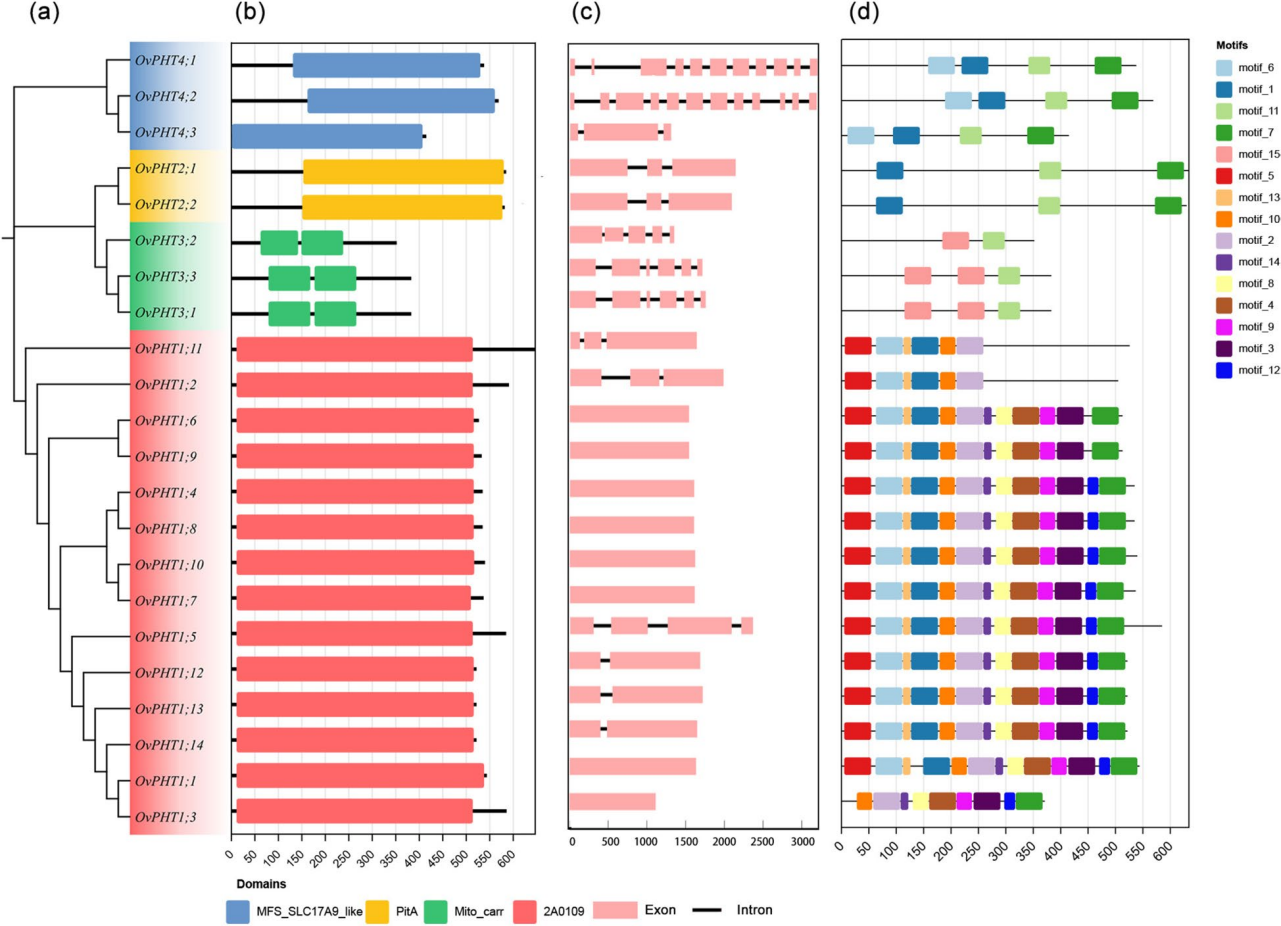
Phylogenetic tree, conserved domain, exon/intron structure and motif of analysis of the PHT genes from *O. violaceus*. **a** The phylogenetic analysis of 22 *OvPHT* genes in *O. violaceus*. **b** Conserved domains of the *OvPHT* genes. **c** Exons/introns of *OvPHT* genes are indicated by pink rectangles, and black lines, respectively. **d** Amino acid motifs in the OvPHT proteins, numbered 1–15, are displayed in different coloured boxes

The intron–exon structure of *OvPHT* genes showed that most *OvPHT1* and *OvPHT2* genes contained 1 ~ 3 exons, with the exception of *OvPHT1;5*, which uniquely possesses 4 exons (Fig. 3c). *OvPHT3* subfamily had 5 ~ 6 exons. Furthermore, OvPHT4 genes had the most exons, such as *OvPHT4;1* and *OvPHT4;1* contained 11 and 12 exons, respectively. It is noteworthy that the exon lengths of *OvPHT3;1* and *OvPHT3;3* were highly conserved, similar gene structure was also found in *OvPHT2;1* and *OvPTH2;2*. Furthermore, we identified and characterized 15 conserved motifs to further elucidate the functional characteristics of OvPHT proteins. The results showed OvPHT1 subfamily members contained more motifs than other PHT subfamilies, and motif 2, 3, 4, 7, 8, 9, 10, 14 were present in all OvPHT1 members (Fig. 3d, Table S2). In addition, all OvPHT proteins, except OvPHT1;3, OvPHT3;1, OvPHT3;2, OvPHT3;3, had motif 1. Motif 11 was found in OvPHT2, OvPHT3 and OvPHT4 subfamilies, but not in OvPHT1. Although OvPHT3 subfamily members contained two to three motifs, motif 15 was present only in this subfamily, underscoring its unique functional properties. Combining the characteristics of conserved domain, motif and intron–exon structure of the OvPHTs, we further confirmed the reliability of the phylogenetic analysis and clustering in *O. violaceus*.

### Collinearity analysis of PHTs between O. violaceus and other plants

Investigating gene duplication events is crucial for understanding the expansion of PHT gene families in *O. violaceus*. As shown in Table 1, nine segmental duplications and two tandem duplications were identified in the *OvPHT* gene family, implying that segmental duplication has a dominant contribution to the expansion of *OvPHT* gene family. The *Ka/Ks* ratio was calculated to further estimate the evolutionary selection pressure on duplication gene pairs. Except for two duplication gene pairs (*OvPHT1;3*; *OvPHT1;11*) and (*OvPHT1;11*; *OvPHT1;12*), for which the *Ka/Ks* ratio was not available, the Ka/Ks values of segmental and tandem duplicated gene pairs ranged from 0.03 to 0.1921, suggesting that *OvPHT* genes underwent purifying selection throughout their evolutionary history (Table 1).

**Table 1:** The *Ka* and *Ks* values of duplicated *OvPHTs* gene pairs

Gene pair name		*Ka*	*Ks*	*Ka/Ks*	Duplicate type
*OvPHT1;4*	*OvPHT1;1*	0.0126	0.1401	0.0899	Segmental duplication
*OvPHT1;4*	*OvPHT1;8*	0.0127	0.3517	0.0363	Segmental duplication
*OvPHT1;3*	*OvPHT1;11*	0.0134	Na	Na	Segmental duplication
*OvPHT1;7*	*OvPHT1;4*	0.0110	0.3671	0.0300	Segmental duplication
*OvPHT1;7*	*OvPHT1;8*	0.0562	1.1998	0.0468	Segmental duplication
*OvPHT1;7*	*OvPHT1;10*	0.0559	1.3275	0.0421	Segmental duplication
*OvPHT2;1*	*OvPHT2;2*	0.0254	0.2216	0.1147	Segmental duplication
*OvPHT3;3*	*OvPHT3;1*	0.0289	0.3504	0.0827	Segmental duplication
*OvPHT4;3*	*OvPHT4;2*	0.0201	0.1045	0.1921	Segmental duplication
*OvPHT1;13*	*OvPHT1;14*	0.0110	0.3671	0.0300	Tandem duplication
*OvPHT1;11*	*OvPHT1;12*	0.0103	Na	Na	Tandem duplication

*Ka*, non-synonymous substitution rate; *Ks*, synonymous substitution rate; *Ka/Ks*, selection pressure ratio; Na, no result

**Fig. 4:**
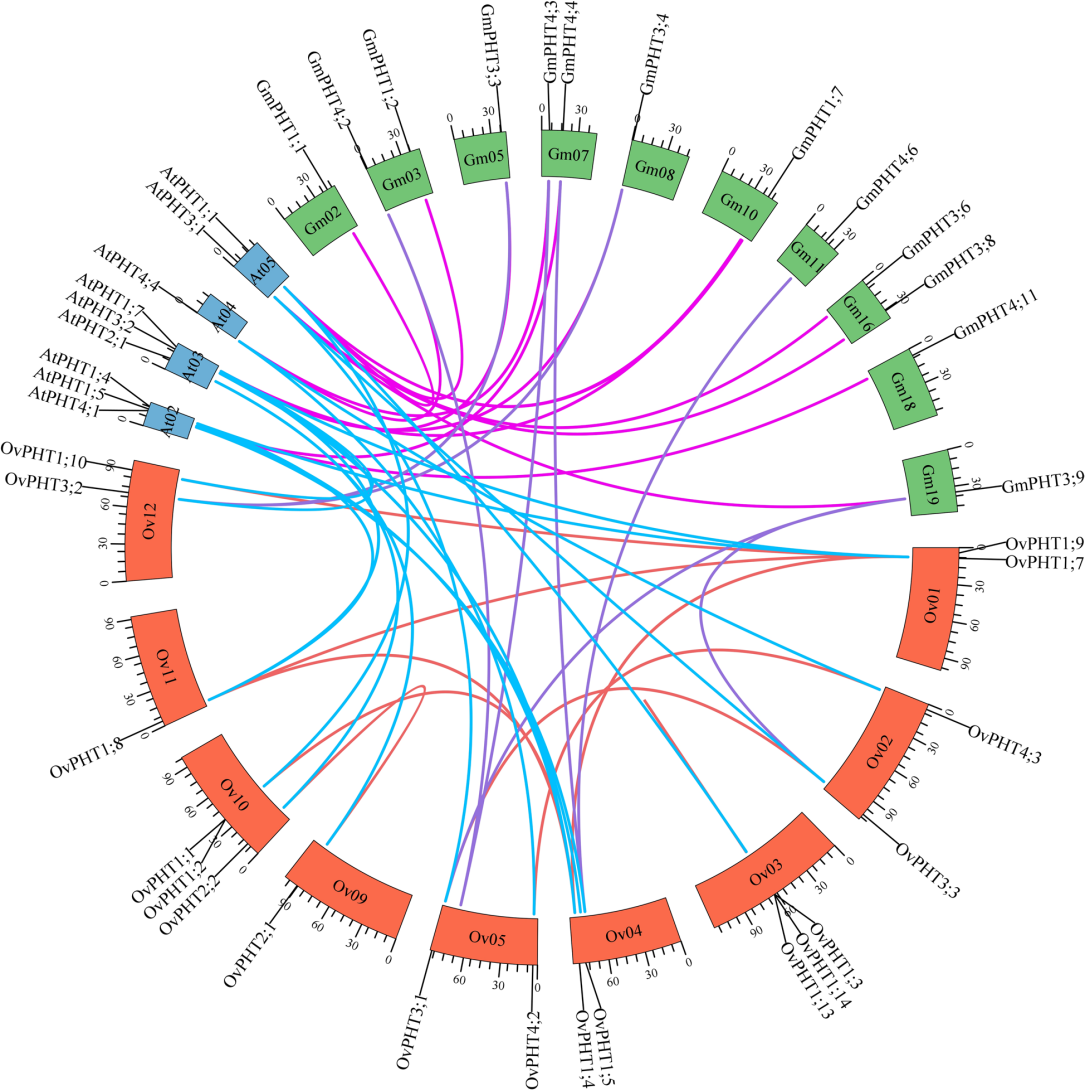
Synteny analysis of *PHT* genes in *A. thaliana*, *G. max* and *O. violaceus*. The red lines indicate the duplicated *OvPHT* gene pairs in the *O. violaceus* genome, blue and purple lines indicate the duplication pairs of *PHT* genes within the genome of *O. violaceus* and *A. thaliana* and *G. max,* respectively. Pink lines indicate the duplication pairs of *PHT* genes within the genome of *A. thaliana* and *G. max*

In addition, to better understand the evolutionary relationships among PHT genes across different plant species, we conducted a collinearity analysis comparing *OvPHT* genes with their homologues in *A. thaliana* and *G. max*. Our findings revealed that the highest number of collinear gene pairs (18 pairs) was observed between *O. violaceus* and *A. thaliana*, which can be attributed to the close evolutionary relationship between these two species (Fig. 4). Additionally, 14 collinear gene pairs were identified between *A. thaliana* and *G. max*, 8 pairs between *O. violaceus* and *G. max*. Notably, the majority of these collinear gene pairs belonged to the same subfamily, while OvPHT1;9 had two homologous genes from PHT4 subfamily in *G. max*, including GmPHT4;4 and GmPHT4;6, indicating that the GmPHT4 subfamily has undergone an increased functional diversity during the whole genome duplication event in *G. max*.

### Cis-elements analysis of the OvPHTs promoter

**Fig. 5:**
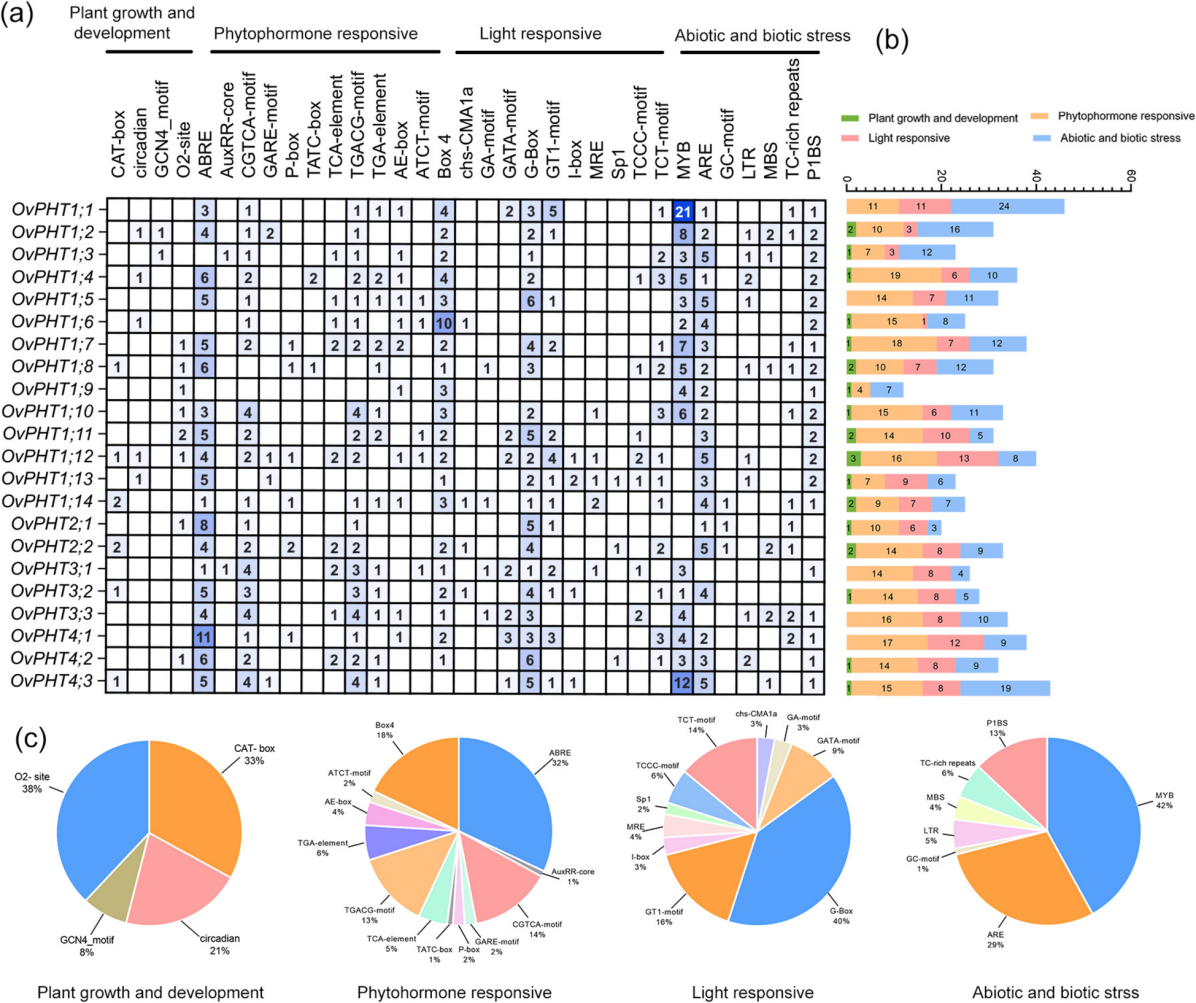
*Cis*-element analysis of the promoter of *OvPHTs*. **a**, **b** Four categories and numbers of *cis*-elements in the *OvPHTs*. **c** Pie charts of each promoter element in different category

We analysed *cis*-elements of the 22 *OvPHT* genes to further investigate the molecular functions (Fig. 5). A total of four types of cis-element were identified, including plant growth and development, phytohormone responsive, light responsive, and abiotic/biotic stress (Fig. 5). The plant growth and development elements were composed of zein metabolism regulation (O2-site, 38%), meristem expression (CAT-box, 33%), circadian rhythm (21%), and endosperm expression regulation (GCN4_motif, 8%). Among the phytohormone responsive, four cis-elements, ABA-related elements (ABRE), Box 4, MeJA responsive (CGTCA-motif and TGACG-motif), auxin responsive (TGA-element and AuxRR-core), accounting for more than 80%. The most abundant cis-element of light responsive was G-Box (40%), followed by GT1-motif (16%), TCT-motif (14%), GATA-motif (9%), and TCCC-motif (6%). All of the *OvPHT* gene promoter regions contained at least one abiotic/biotic stress element, the most abundant types were MYB (42%) and anaerobic induction (ARE, 29%) elements. In addition, it should be noted that the low phosphorus response cis-element P1BS was observed in all members of the OvPHT1 and OvPHT4 subfamilies, while 2 of the 3 OvPHT3 genes have 1 P1BS element. But no P1BS element was present in the promoters of OvPHT2 genes, and we also observed significant variation in the number of cis-elements present within the same subfamily. For instance, the promoter of *OvPHT2;2* contained 48 cis-elements, which was significantly higher than that observed in the promoter of *OvPHT2;1*(20).

### Tissue-specific expression of OvPHTs

**Fig. 6:**
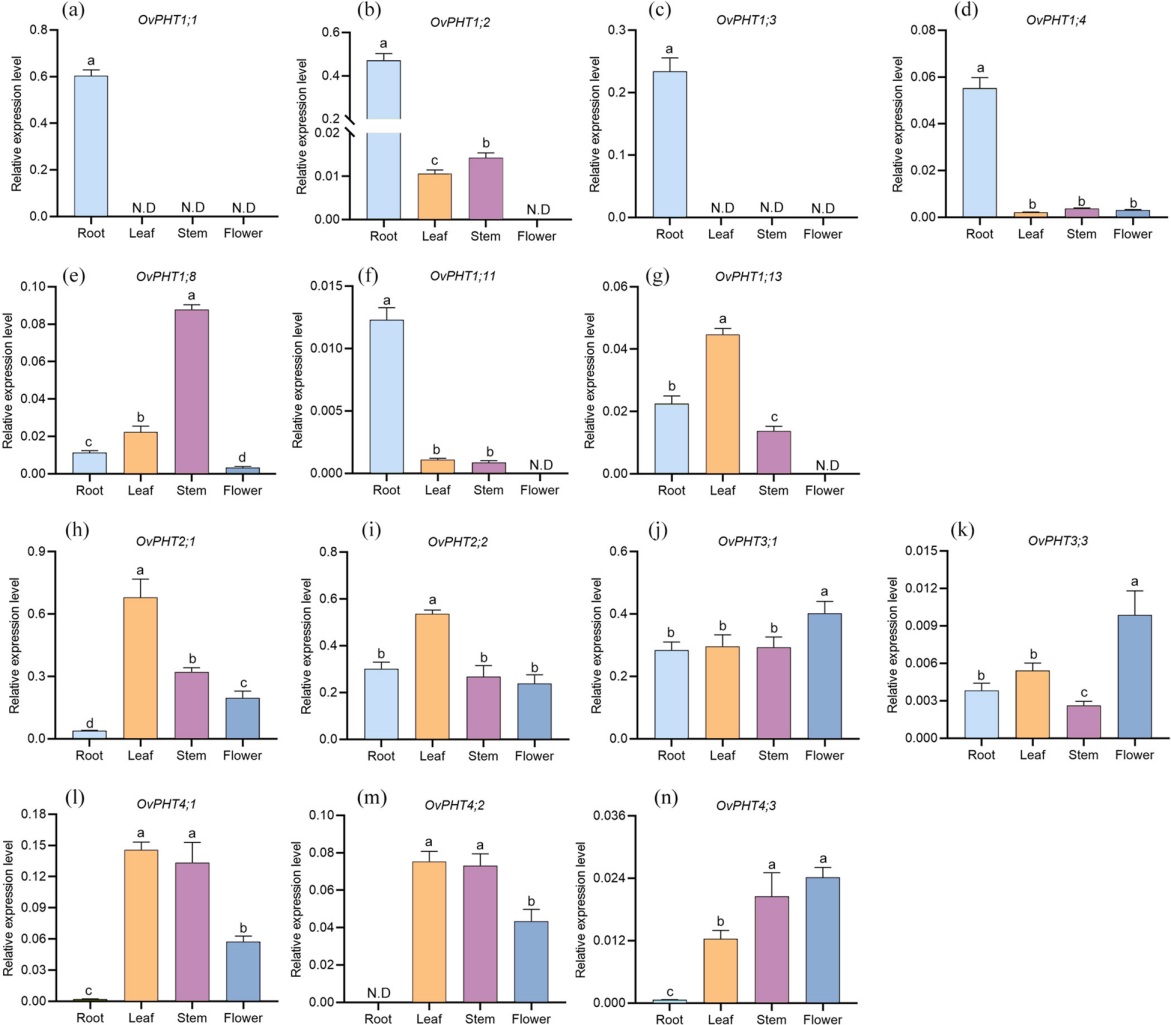
Expression patterns of *OvPHTs* in different tissues of *O. violaceus*. One-way ANOVA was used for the statistical analysis (*P* < 0.05)

In this study, to explore the possible functions of OvPHTs in *O. violaceus* development, we analyzed the tissue-specific expression patterns of *OvPHTs *in roots, stems, leaves, and flowers using RT-PCR. Except for *OvPHT1;5/1;6/1;7/1;9/1;10/1;12/1;14* and *OvPHT3;2*, whose expression was not detectable in the four tissues examined, the transcripts of the remaining *PHTs* could be detected in at least one of the selected tissues, and the expression pattern exhibited distinct but partially overlapping patterns (Fig. 6).

*OvPHT1;4* and *OvPHT1;11* exhibited high expression levels in the roots While *OvPHT1;8* and *OvPHT1;13* were abundantly expressed in stems and leaves, respectively. Besides, the transcripts of *OvPHT1;1* and *OvPHT1;3* only could be detected in roots. Both *OvPHT2;1* and *OvPHT2;2* were observed to be expressed in all our tissues, with a relatively higher expression in leaves. Notably, *OvPHT2;2* has a higher expression level in roots compared to *OvPHT2;1*. Two *OvPHT3* subfamily members, *OvPHT3;1* and *OvPHT3;3* showed ubiquitously expressed in roots, leaves, stems, and flowers. Additionally, the expression level of *OvPHT3;3* was higher than that of *OvPHT3;1* in flowers. It is noteworthy that the transcripts of all *OvPHT4* genes were detected in leaves, stems, and flowers, but were barely detectable in roots, which suggested that *OvPHT4* genes might be primarily involved in P redistribution rather than uptake. In addition, the strikingly similar expression patterns observed between *OvPHT4;1* and *OvPHT4;2* imply that these two members might function redundantly in Pi utilization.

### Effect of low-Pi stress on the expression of OvPHTs

**Fig. 7:**
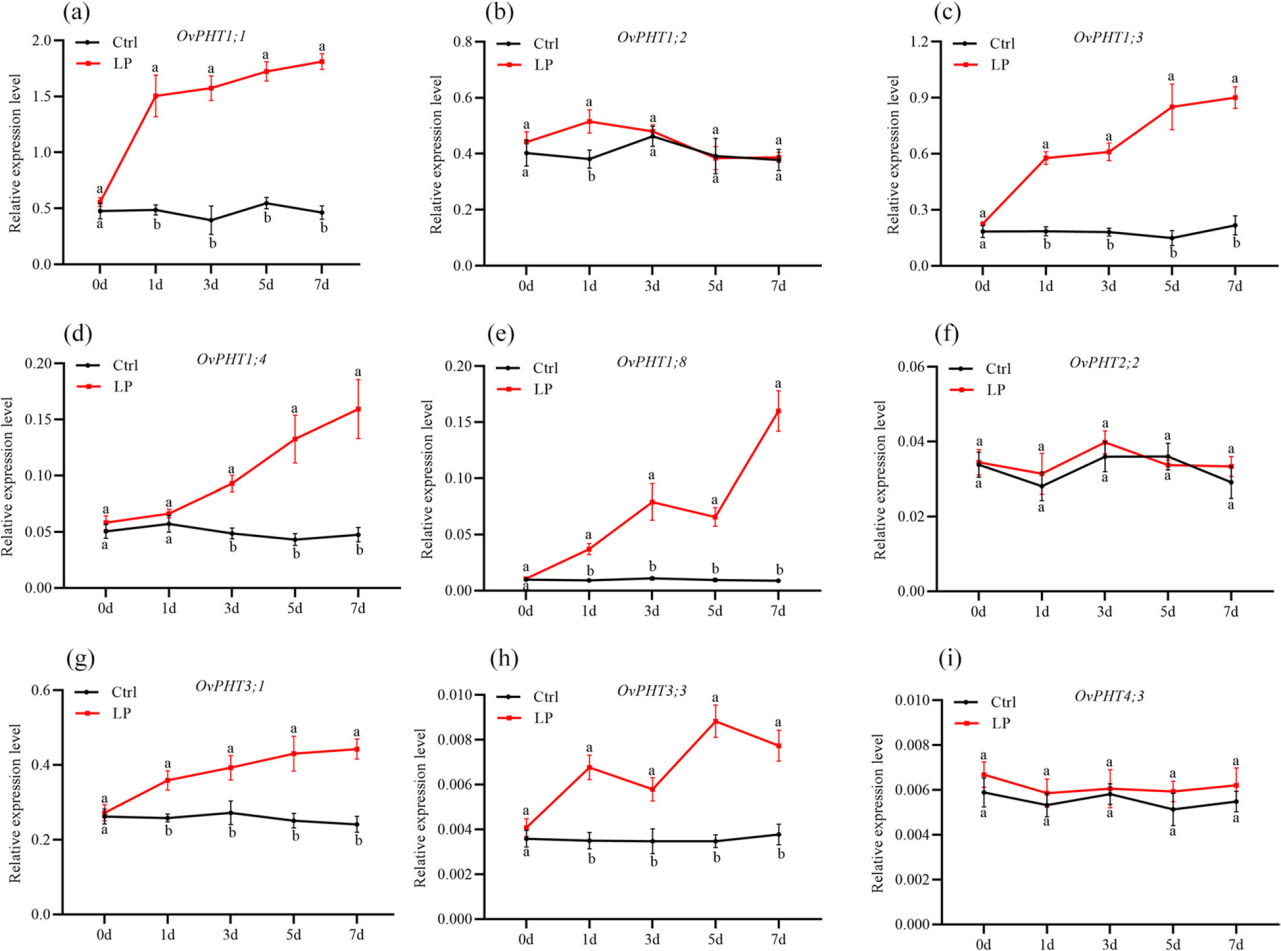
Expression profiles of *OvPHTs* in the roots under low P stress. Ctrl: 90 μmol P, normal P condition; LP: 1 μmol P, P deficiency. Error bar represents SE of three independent biological replicated. Student’s *t*-test was used for the statistical analysis (*P* < 0.05)

It has been repeatedly documented that the PHT transporters play an irreplaceable role in phosphate uptake and translocation, especially under P-deficient conditions. To explore the potential transcriptional responses of *OvPHTs*, the time-course expression patterns of 12 selected *OvPHTs* from four subfamilies (including *OvPHT1;1/1;2/1;3/1;4/1;8*, *OvPHT2;2*, *OvPHT3;1/3;3* and *OvPHT4;3*) were examined in the roots of *O. violaceus* seedings cultured under P-sufficient and P-deficient conditions. As shown in Fig. 7, with the exception of *OvPHT2;2* and *OvPHT4;3*, whose transcript levels remained unchanged under varying phosphate conditions, the remaining 10 *OvPHT* members exhibited significant induction in response to P deficiency. The transcript levels of *OvPHT1;1* were notably increased after 1-day P-deficient treatment. *OvPHT1;2* showed only slightly but significantly up-regulated by a 1-days P-deficient treatment. The expression patterns of *OvPHT1;3* and *OvPHT3;1* in response to P starvation exhibited some similarities. Both genes showed a significant increase in transcript levels under P-deficient treatment from day one, with a continuous enhancement that peaking after 7 days. In contrast to these rapidly responding OvPHTs, the expression level of *OvPHT1;4* was significantly induced only after 3 days of low-P treatment, while *OvPHT3;3* was up-regulated at all time points, but the transcript levels were slightly decreased at 3 and 7 days. Similarly, the transcripts of *OvPHT1;8* were also significantly induced by low-P treatment, with a sharp increase in expression reaching its maximum at 7 days.

### Localization and functional characterization of OvPHT1;1

**Fig. 8:**
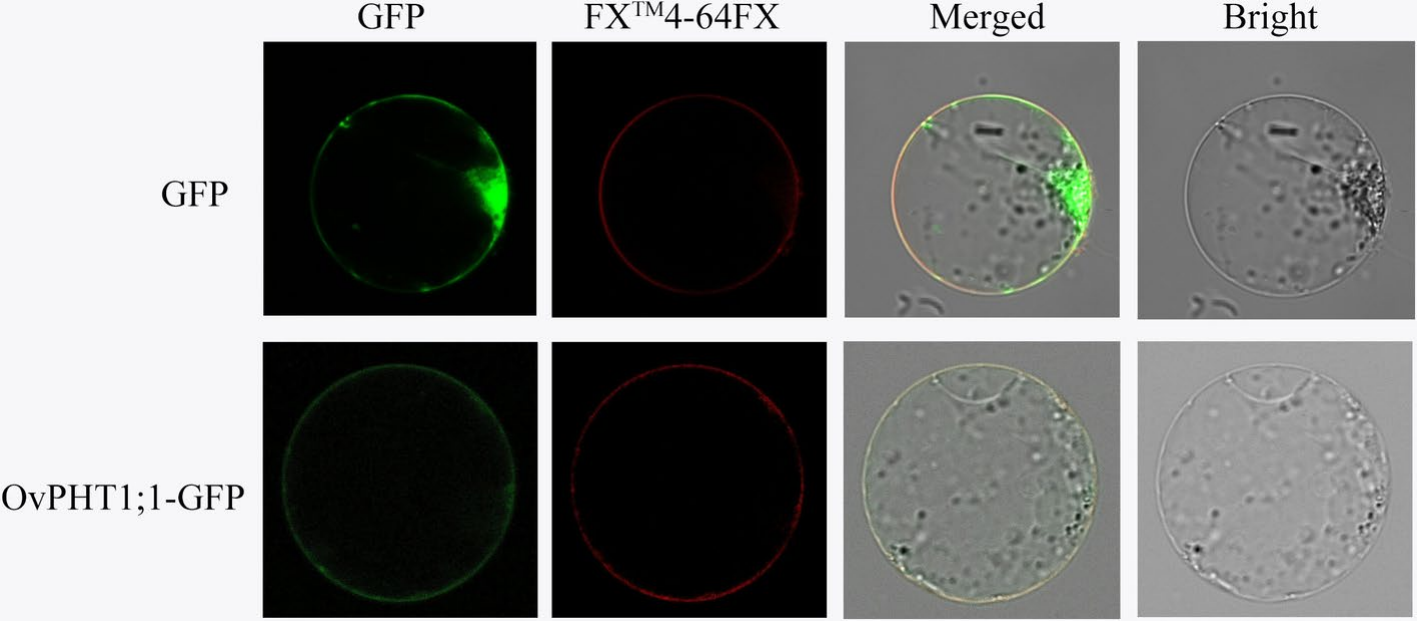
Subcellular localization of OvPHT1;1 in the rice protoplast. Confocal laser scanning microscopy images of 35S::eGFP (**a**) and 35S::OvPHT1;1-eGFP (**b**). FM4-64FX: a plasma membrane-specific dye. Bars = 20 μm

Since the OvPHT1;1 only expressed in in roots, and had a higher expression level than other OvPHTs, we selected OvPHT1;1 for further functional characterization. To determine whether OvPHT1;1 functions as a plasma membrane-localized protein, its subcellular localization was investigated..Microscopic visualization showed that the 35S::OvPHT1;1::GFP recombinant protein localized to the plasma membrane (Fig. 8), which was consistent with the bioinformatic predictions.

**Fig. 9:**
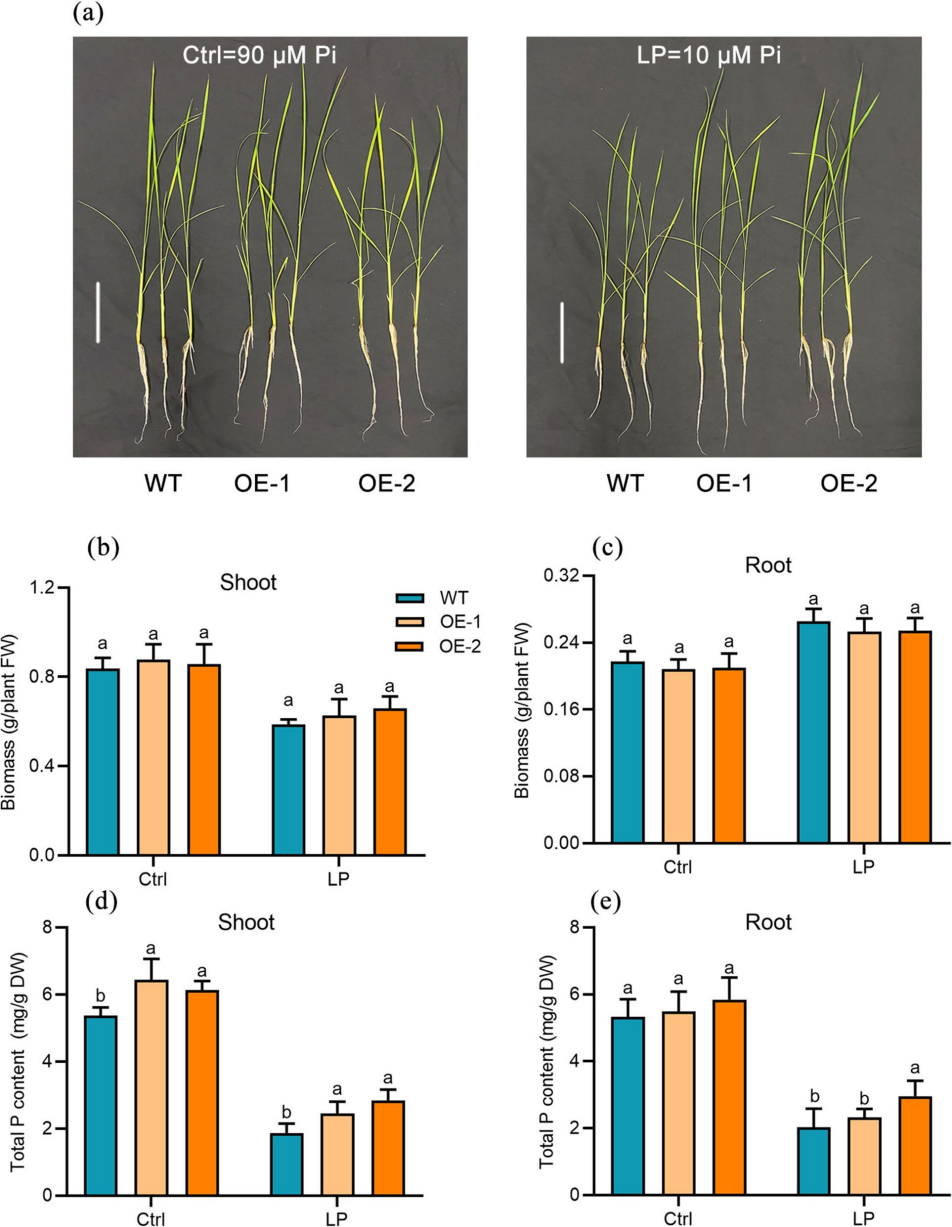
OvPHT1;1 mediates phosphate accumulation in transgenic rice plants. **a**, **b** Phenotype of OvPHT1;1 overexpression lines and WT plants grown under Ctrl (90 μmol P) and LP (1 μmol P) hydroponic conditions. The biomass (**c**, **d**) and total P content (**e**, **f**) of shoots and roots of WT plants and overexpression lines. Error bar indicate ± SE (*n* = 6). Student’s *t*-test was used for the statistical analysis (*P* < 0.05)

To further confirm the impact of increased expression of OvPHT1;1 on phosphate uptake, we further transformed the 35S-OvPHT1;1 chimeric gene into rice plants. Constitutive expression OvPHT1;1 in rice appeared to have no visible effect on plant growth under either of the two P conditions (Fig. 9). However, a 20% increase of P accumulation could be detected in both shoots and roots of the 35S-OvPHT1;1 transgenic rice plants compared to WT plants under the low-P condition. These results indicate that constitutive overexpression of OvPHT1;1 in planta might be able to improve P uptake particularly under Pi-deficient conditions.

## Discussion

Phosphate is a crucial component involved in various biological processes, such as energy metabolism, the formation of vital macromolecules, and photosynthesis [2]. As the most important transporter for plants acquire phosphate from soil solution, PHT family genes have been identified in many plant species. Benefiting from the availability of high-quality genomic data, our study has identified 22 genes encoding *OvPHT* transporters in *O. violaceus*. Among the PHT families in *O. violaceus*, a total of 22 *OvPHT* genes were divided into four subfamilies, with *OvPHT1*, *OvPHT2*, *OvPHT3* and *OvPHT4* having fourteen, two, three and three members, respectively. This subfamily distribution closely align with that observed in other high plants [15]. Notably, unlike Arabidopsis and rice, which contain single genes in the PHT2 subfamily, two *OvPHT* genes (*OvPHT2;1* and *OvPHT2;2*) were classified into the PHT2 subfamily. The chromosome localization of the 22 *OvPHT* genes exhibited a similar distribution pattern to that of AtPHTs and OvPHTs, displaying an uneven distribution across 9 chromosomes. Additionally, the aliphatic indices of these 22 OvPHTs were positive, with pI values exceeding 7, indicating that the proteins encoded by the *OvPHT* genes possess hydrophobic characteristics, resulting in relatively low solubility of their primary structures in neutral solutions [42]. Generally, an instability index below 40 suggests a relatively stable protein primary structure[43, 44]. we observed that the instability indices of members of the *OvPHT1* and *OvPHT2* subfamilies were below 40, with the exception of *OvPHT1;9*. Conversely, almost all of the *OvPHT3* and *OvPHT4* genes exhibited instability indices exceeding 40. With the exception of *OvPHT3* genes, which either lacked TMS or contained only one, the remaining *OvPHT* genes possessed TMS counts ranging from 10 to 12, this result also be found in rice and Arabidopsis [45–47]. It has been reported that the PHT1 proteins contained a conserved domain (GGDYPLSATLxSE) [48]. Consistent with this, we found this conserved domain in all of the *OvPHT1* genes. In rice, *OsPT8* expression was up-regulated by low-P treatment and mediated phosphate uptake under P-deficient conditions, while OsPT8^S517^ could be phosphorylated and resulted in reduced phosphate uptake under P-sufficient conditions [49]. Interestingly, a series of similar location of serine were exhibited in OvPHT1;5, OvPHT1;6 and OvPHT1;8, indicating that the mechanism of posttranslational regulation may also be conserved in OvPHT1 genes. Previous research on Arabidopsis, rice, wheat and potato have shown that the PHT2 subgroup only contains a single member, which play a key role in phosphate utilization [26–28, 50]. Different from this plants, two OvPHT2 genes were identified in *O. violaceus* genomic. Interestingly, Notably, while *OvPHT2;1 and OvPHT2;2* share high amino acid sequence identity, their promoter regions contain significantly different numbers of stress- and phytohormone-related *cis*-elements. This divergence in regulatory elements suggests that these paralogs likely perform distinct functions in response to environmental stresses and phytohormonal signals.

It is generally accepted that *cis*-elements control the efficiency of promoters and regulate the tissue-specific or development-dependent expression. The promoter regions of OvPHTs contain several types of *cis*-elements, such as plant growth and development-related, light-related, hormone-related, as well as biotic/abiotic response-associated elements [51–53]. These results indicated that OvPHTs may play an important role in various stress responses. The P1BS *cis*-element has been extensively documented as a crucial regulator of the expression of numerous P starvation-induced (PSI) genes, interacting with the MYB transcription factor PHR1 under low-P conditions [52, 54, 55]. In our study, the P1BS *cis*-element was found in 19 out of the 22 OvPHTs promoters, among which OvPHT1 and OvPHT4 members contained at least one P1BS element, suggesting their potential role in a conserved signaling pathway for the P starvation responses (PSRs) in plants. Recently, several studies revealed that PHT might be involved in response to drought stress. In *Brassica napus*, the transcription of 18 PHT genes was increased in leaves under drought stress, and 10 members were downregulated in roots [56]. The phosphate level also influenced the transcription of PtPHT genes under drought stress in Poplar. For example, the expression levels of *PtPHT3.3b*, *PtPHT4.3* and *PtPHT4.5a* were significantly reduced by drought under high-P conditions, whereas this inhibitory effect was greatly weakened under low-P conditions [57]. In *O. violaceus*, the drought-inducing cis-element MBS was also observed in seven PHT genes. With the exception of *OvPHT2;2*, the other six genes also contained P1BS element.

It has been established that evolutionary events, such as whole-genome duplication, tandem duplications and segmental duplications, lead to chromosome rearrangement, gene family expansion or loss, and function divergence. Chen et al. demonstrated that tandem duplications might be major evolutionary mechanisms for increasing the members of *SlPT* genes [48]. In our study, collinearity analysis of *OvPHT* genes revealed a higher frequency of segmental duplication events (9 gene pairs) compared to tandem duplications (2 gene pairs). This inconsistency might be explained by species differences.

In addition, except for two gene pairs, *OvPHT1;3/OvPHT1;11* and *OvPHT1;11/OvPHT1;12*, which were non *Ka/Ks* ratio value, the remaining gene pairs had *Ka/Ks* ratio less than 1, indicating that the OvPHT genes underwent purifying selection for retention during evolution. Compared with grape genome, *O. violaceus* experienced two whole-genome duplication (WGD) events, including Brassicaceae-specific α-WGD event and meso-tetraploidization event. It was estimated that 15 grape genes would correspond to 60 ortholog genes in *O. violaceus*, whereas due to the strong purifying selection leading to the gene loss or translocation, only 22 *OvPHT* genes were ultimately retained in *O. violaceus* [34, 38]. Previous study has revealed that the segmental duplication could accelerate the process of intron loss rather than intron gain [58]. In *O. violaceus*,the intron numbers in *OvPHT3* and *OvPHT4* genes were greater than those in *OvPHT1* genes, suggesting that the ancestral genes might have belonged to the *PHT3* and *PHT4* subfamilies. Notably, this conclusion is further supported by the absence of PHT genes in the PHT1 or PHT2 subfamilies in two ancient species, *V. carteri* and *C. reinhardtii* [59]. The exon lengths of *OvPHT3;1* and *OvPHT3;3* were highly conserved, mirroring the structural conservation between *OvPHT2;1* and *OvPHT2;2*, suggesting a shared evolutionary constraint across PHT subfamilies.

Gene duplication often precedes functional differentiation, offering plants a diverse array of strategies to adapt to external environmental stresses. As the largest subfamily, the PHT1 family plays a dominant role in plant P acquisition from low-P soil solution. As excepted, most PHT1 genes exhibit Pi starvation-induced expression and are predominantly expressed in the roots' nutrient uptake zones, such as lateral roots, root hairs, and epidermal cells [18, 23, 24, 60]. In our study, an analysis of the tissue-specific and low-P-induced expression patterns of *OvPHT* genes revealed that *OvPHT1;1* and *OvPHT1;3* are specifically expressed in *O. violaceus* plant roots and are significantly upregulated by low-P treatments. Similarly, *SlPT2* and *SlPT6*, orthologs of *OvPHT1;1* and *OvPHT1;3*, respectively, also show predominantly expressed in the roots and are induced under low-P conditions. Furthermore, OvPHT1;1 overexpression in rice increased P uptake under low-P conditions. These findings indicate that these two *OvPHT* genes are crucial for Pi uptake, particularly under P-deficient conditions. The maintenance of Pi homeostasis in plant cells is not only regulated by plasma membrane-localized phosphate transporters, but also relies on chloroplasts-, vacuole-, mitochondria- and Golgi-located Pi transporters. In Arabidopsis, *AtPHT2;1* encodes a chloroplast-localized phosphate transporter that mediates phosphate intracellular trafficking [26]. Knock-down of TaPHT2;1 could significantly decreases phosphate accumulation in the chloroplast in both of P-deficient and P-sufficient conditions [50]. In this study, higher levels of the two *OvPHT2* gene transcripts were detected in leaves than in other tissues, similar to the expression patterns of *OsPHT2;1* in rice. It has been well documented that P-deficient can stimulate flavonoid biosynthesis in vascular plants [61–63]. The mutant of *ospht2;1* caused a significant decrease of flavonoids contents under P sufficiency and deficiency [28]. *OvPHT3;3* and its Arabidopsis ortholog, *AtPHT3;3* exhibited comparable expression patterns in floral organs, suggesting a potential role of *OvPHT3;3* in reproductive development. In Arabidopsis and pepper, most PHT4 members showed higher transcript levels in leaves than in roots. In our study, the expression of OvPHT4 genes was primarily detected in shoots but not in roots These converging findings suggest that *OvPHT4* genes may function primarily as mediators of phosphate translocation rather than uptake in *O. violaceus*.

Taken together, a comprehensive characterization of the *PHT* gene family in *O. violaceus*, including evolutionary and expression patterns, was analyzed in the present study. A total of 22 *OvPHT* genes were identified and divided into four distinct subfamilies. Notably, proteins within the same subfamily exhibit remarkable conservation in terms of physicochemical properties, gene structures, and motif compositions. Synteny analysis further revealed that the *OvPHT* gene family underwent 7 segmental and 2 tandem duplication events, which were subsequently subjected to purifying selection pressure during their evolution. Furthermore, the expression patterns of *OvPHT* genes in various tissues and P-starvation conditions indicated that *OvPHT* genes perform important functions in phosphate homeostasis. Thus, these findings not only enhance our understanding of the evolutionary and expression patterns of *OvPHTs* but also provide promising candidate *OvPHT* genes for molecular breeding of *O. violaceus* with high Pi acquisition and utilization efficiency.

## Materials and methods

### Plant materials, growth and treatments

The seeds of *O. violaceus* (obtained from Chinese Academy of Agricultural Sciences) were initially surface-sterilized and germinated using moist filter paper. Subsequently, they were cultured in a climate chamber at the College of Resource and Environment, Anhui Science and Technology University (Fengyang, China), and maintained at 22℃with long-day conditions (16 h of Light and 8 h of dark). For P-deficient hydroponic experiments, the seedlings were transferred to the nutrient solution containing either 1 μmol P (P-deficient) or 90 μmol P (P-sufficient). The nutrient solution was refreshed every 3 days. For tissue-specific expression analysis, the leaves, roots, stems and flowers (the plants are in a natural growth state) were sampled separately and immediately frozen in liquid nitrogen. All the collected samples were stored at −80℃ to facilitate RNA extraction. Three independent biological replicates were included in each experiment.

### Identification and sequence analysis of OvPHT genes

The whole-genome sequence of *O. violaceus* was downloaded from http://www.bioinformaticslab.cn/pubs/OV_data/. The OvPHT transporters were identified according to the AtPHT genes by BLASTP search method against the *O. violaceus* genome. Candidate OvPHT genes were selected based on amino acid sequences sharing over 50% similarity and an E-value threshold of 1.0E^−10^. The HMMER (version 3.2.1) was used to further search OvPHT genes in the whole genome, based on the PHT transporter domains including PF00083, PF01384, PF00153 and PF07690 in Pfam (http://pfam.xfam.org/). The protein sequences and chromosomal location of OvPHT genes were obtained from the reference genome of *O. violaceus*. Using the ExPASy online software (http://web.expasy.org/protparam/), we gathered crucial information, including molecular weight, pI, instability/aliphatic index, and grand average of hydropathicity. Moreover, the subcellular locations of the OvPHT proteins were predicted using online tools of WOLF PSORT (http://wolfpsort.hgc.jp/) and CELLO (http://cello.life.nctu.tw//).

### Phylogenetic analysis of OvPHT genes

Multiple sequence alignments of PHT proteins, including OvPHTs, AtPHTs, OsPHTs and GmPHTs were performed to explore the phylogenetic relationship using ClustalW tool within MEGA-X. Phylogenetic tree was then constructed with MEGA 7.0 with Neighbor-Joining method based on the aligned sequences [64]. The parameters for this analysis were set as described in detail by Liu et al. [65]. The online software TIMETREE (http://www.timetree.org/) was used to construct a species tree.

### Gene structure and conserved domain analysis

The exon–intron structure of OvPHT genes were drawn using the Gene Structure Display Server (http://gsds.cbi.pku.edu.cn). To identify conserved motifs, the MEME v5.5.5 online program (https://meme-suite.org/meme/tools/meme) was employed to elucidate conserved motifs with the number set to 15. For cis-element analysis, a 2000-bp nucleotide sequences upstream of the *OvPHT* genes were uploaded to the PlantCare online software (http://bioinformatics.psb.ugent.be/webtools/plantcare/html/). The cis-elements were classified based on their functions and were visualized using the ChiPlot online software (https://www.chiplot.online/).

### Gene duplication and collinearity analysis

The MCScanx program within the TBtools software was employed to analyze duplication events among the 22 OvPHT genes. The Simple Ka/Ks Calculator tool used to calculate the frequency of synonymous mutation rate (Ks), non-synonymous mutation rate (Ka) and rate of Ka/Ks for each gene pair. The collinearity analysis of *PHT* genes among homologs in Arabidopsis, soybean, and *O. violaceus* was performed using the One-Step MCScanX program in TBtools software (v2.080), with visualization conducted via the Advanced Circos program [66].

### RNA extraction and quantitative real-time PCR

Total RNA was extracted from various plant tissues using the Trizol (Invitrogen, USA) reagent, followed by a quality assessment with the NanoDrop 2000 (Thermo Fisher Scientific, USA). A reverse transcription kit (Takara Biotechnology, Dalian, China) was used to synthesis first-strand cDNA. RT-PCR reactions were set as described by Fang et al. (2023), and then performed on the QuantStudio 6 Flex RealTime PCR System (Thermo Fisher Scientifix, USA). All primers used for RT-PCR are listed in Table S3. OvActin (OV07G00868.1) was used as the reference gene. The relative gene expression level were calculated by the 2^−ΔΔCT^ method. Three technical replicates were performed for RT-PCR analysis.

### Subcellular localization analysis of OvPHT1;1

The coding region of *OvPHT1;1* without the stop codon was ligated into the pRCS2 vector. The plasmids were then transformed into the *Agrobacterium tumefaciens* strain EHA105, and subsequently expressed in rice protoplasts as described by Liu et al. [65]. After 48 to 72 h infiltration, GFP fluorescence was imaged using confocal microscopy (Leica Confocal TCS-SP8).

### Vectors and rice transformation

For the overexpression of *OvPHT1;1* in rice, the coding region of *OvPHT1;1* was amplified from the *O. violaceus* cDNA Libary and then cloned into the pCAMBIA 1300 vector. The vector constructs were transformed into the mature embryos developed from seeds of wild-type plants (cv*. Nipponbare*) via *Agrobacterium tumefaciens*-mediated transformation, as previously described [18]. The WT and transgenic Lines were grown hydroponically in 1 μmol Pi or 90 μmol P conditions. The shoot and root samoles were harvested at 3 weeks for quantification the total P content. For the measurement of total P concentration, the dried samples were digest with 98% H_2_SO_4_ and 30% H_2_O_2_, followed by ICP-MS analysis.

### Statistical analysis

The data means and standard errors (SE) were determined using Microsoft Excel 2019. Statistical significance between different plant genotypes and treatments were analyzed by analysis of variance with IBM SPSS Statistics (version 25.0), followed by Turkey’s test (*P* < 0.05) or Student’s *t*-test (*P* < 0.05). GraphPad Prism 8.0.1 was used for figure drawing [67].

## Supplementary Information

Supplementary Material 1.

Supplementary Material 2.

Supplementary Material 3.

Supplementary Material 4.

## Data Availability

The genome sequence information of the eleven selected species were obtained from the National Center for Biotechnology Information websites. The data sets supporting the conclusions of this study are included within the article and its additional files.
