# Supplementary figures and images for: Genome-wide systematic characterization of PHT gene family and its member involved in phosphate uptake in Orychophragmus violaceus
Source: BMC Genomics. 2025 Sep 29;26:876. doi: 10.1186/s12864-025-12091-x (PMC12482593; doi:10.1186/s12864-025-12091-x)

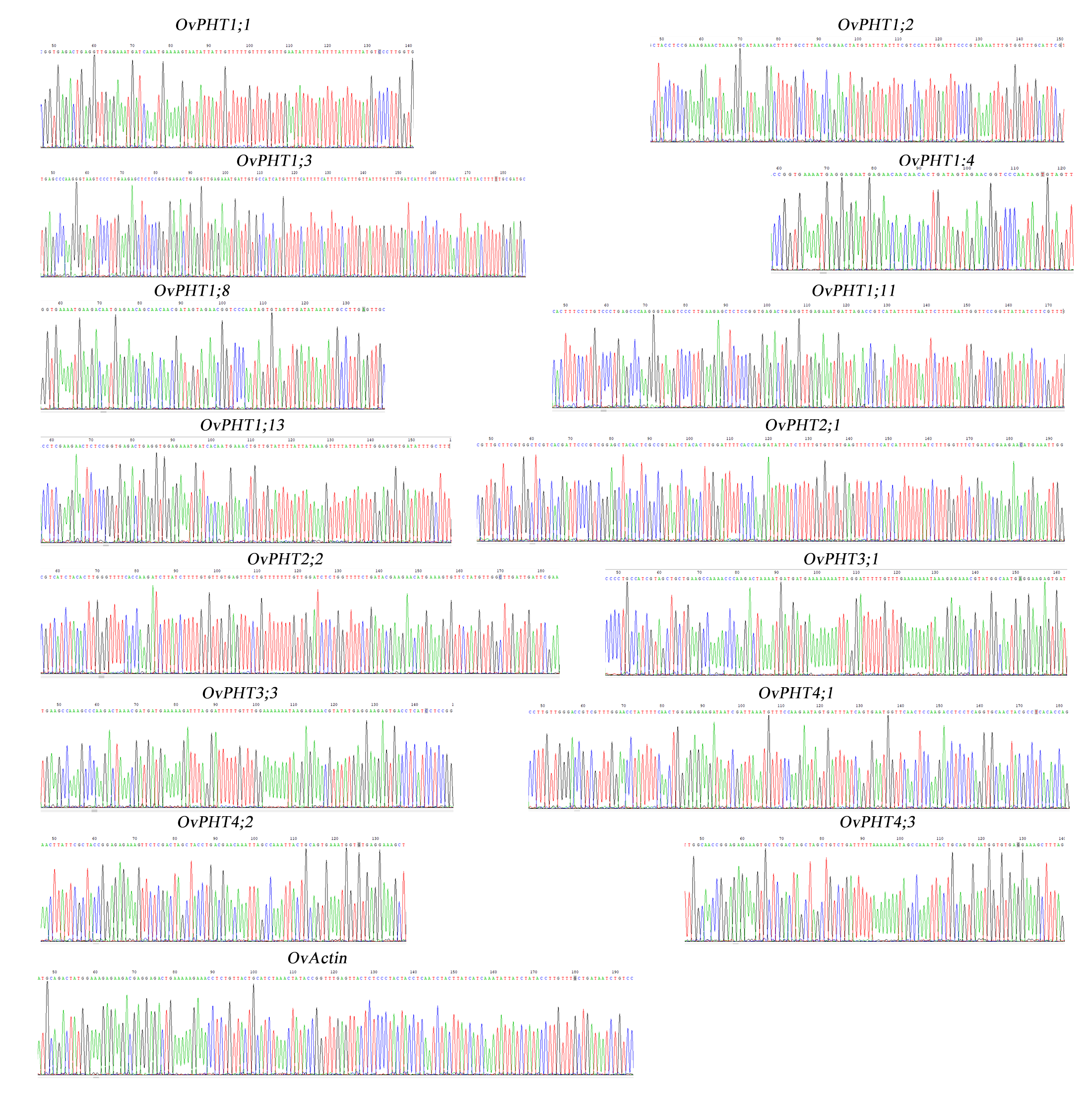

Supplement: Supplementary file 1 — Supplementary Material 1. [file 12864_2025_12091_MOESM1_ESM.tif]
