# Supplementary material for: Genome-wide systematic characterization of PHT gene family and its member involved in phosphate uptake in Orychophragmus violaceus
Source: BMC Genomics. 2025 Sep 29;26:876. doi: 10.1186/s12864-025-12091-x (PMC12482593; doi:10.1186/s12864-025-12091-x)
Supplement: Supplementary file 2 — Supplementary Material 2. [file 12864_2025_12091_MOESM2_ESM.docx]

Table S1*.* Detailed information of all identified *PHT* proteins of *O. violaceus*

| Protein name | Sequence ID | Number of amino acids | Molecular weight  /kDa | pI | Instability index | Aliphatic index | Grand average of hydropathicity | Chromosomal Location | Subcellular location prediction |
| --- | --- | --- | --- | --- | --- | --- | --- | --- | --- |
| *OvPHT1;1* | OV10G026500.2 | 534 | 58.64 | 8.81 | 35.65 | 88.65 | 0.331 | OV10 | PM |
| *OvPHT1;2* | OV10G026510.1 | 521 | 57.36 | 9.19 | 28.33 | 94.99 | 0.422 | OV10 | PM |
| *OvPHT1;3* | OV03G014210.1 | 521 | 57.32 | 9.15 | 29.39 | 94.24 | 0.399 | OV03 | PM |
| *OvPHT1;4* | OV04G007900.1 | 584 | 63.61 | 9.09 | 33.60 | 88.61 | 0.330 | OV04 | PM |
| *OvPHT1;5* | OV04G014770.3 | 512 | 55.88 | 9.04 | 34.50 | 96.07 | 0.471 | OV04 | PM |
| *OvPHT1;6* | OV03G037060.1 | 539 | 59.01 | 8.82 | 34.17 | 89.65 | 0.357 | OV03 | PM |
| *OvPHT1;7* | OV01G008340.1 | 534 | 58.56 | 8.91 | 36.60 | 90.49 | 0.349 | OV01 | PM |
| *OvPHT1;8* | OV11G006890.1 | 512 | 36.43 | 9.14 | 33.24 | 95.90 | 0.457 | OV11 | PM |
| *OvPHT1;9* | OV01G005350.1 | 489 | 54.53 | 9.74 | 59.40 | 98.61 | 0.018 | OV01 | Nuclear |
| *OvPHT1;10* | OV12G033180.1 | 536 | 58.32 | 8.80 | 30.02 | 87.78 | 0.345 | OV12 | PM |
| *OvPHT1;11* | OV10G026440.1 | 521 | 57.30 | 9.19 | 28.63 | 94.05 | 0.410 | OV10 | PM |
| *OvPHT1;12* | OV10G026430.1 | 543 | 60.11 | 9.32 | 30.10 | 96.17 | 0.412 | OV10 | PM |
| *OvPHT1;13* | OV03G014040.1 | 505 | 57.15 | 9.45 | 39.34 | 87.86 | 0.001 | OV03 | PM |
| *OvPHT1;14* | OV03G014050.1 | 370 | 41.08 | 9.15 | 34.22 | 90.00 | 0.326 | OV03 | PM |
| *OvPHT2;1* | OV09G016070.1 | 584 | 60.97 | 9.43 | 25.84 | 101.47 | 0.567 | OV09 | PM |
| *OvPHT2;2* | OV10G036570.1 | 581 | 60.75 | 9.53 | 29.19 | 99.48 | 0.516 | OV10 | PM |
| *OvPHT3;1* | OV05G034450.1 | 351 | 37.44 | 9.29 | 37.05 | 92.28 | 0.180 | OV05 | Chloroplast |
| *OvPHT3;2* | OVunG018830.1 | 382 | 40.88 | 9.23 | 44.65 | 82.51 | 0.159 | OV12 | Chloroplast |
| *OvPHT3;3* | OV02G008140.1 | 382 | 40.91 | 9.17 | 41.54 | 82.04 | 0.145 | OV02 | Chloroplast |
| *OvPHT4;1* | OV04G018350.1 | 537 | 59.59 | 9.04 | 47.99 | 102.53 | 0.331 | OV04 | Nuclear |
| *OvPHT4;2* | OV05G000500.1 | 568 | 62.74 | 9.37 | 43.41 | 99.70 | 0.291 | OV05 | PM |
| *OvPHT4;3* | OV02G039060.1 | 414 | 45.41 | 9.16 | 45.03 | 107.85 | 0.580 | OV02 | PM |
