# Supplementary material for: Genome-wide systematic characterization of PHT gene family and its member involved in phosphate uptake in Orychophragmus violaceus
Source: BMC Genomics. 2025 Sep 29;26:876. doi: 10.1186/s12864-025-12091-x (PMC12482593; doi:10.1186/s12864-025-12091-x)
Supplement: Supplementary file 3 — Supplementary Material 3. [file 12864_2025_12091_MOESM3_ESM.docx]

Table S2 Conserved motifs identified from the *OvPHT* genes

| Motif 1 |  |
| --- | --- |
|  | MTTLCFFRFWLGFGIGGDYPLSATIMSEYANKKTRGAFIAAVFAMQGFGI |
| Motif 2 |  |
|  | PQADYVWRIILMVGALPAALTYYWRMKMPETARYTALVAKNIKQAAQDMS |
| Motif 3 |  |
|  | PYNHWIHPDNRIGFVVMYDLTFFFSNFGPNSTTFIVPSEIFPSRLRSTCH |
| Motif 4 |  |
|  | FYSQNLFQKDIFSAIGWIPPAATMNAIHEVFKIARAQTLIALCSTVPGYW |
| Motif 5 |  |
|  | QLGVLKALDVAKTQLYHFTAIVIAGMGFFTDAYDLFCISLVTKLLGRIYY |
| Motif 6 |  |
|  | PGTLPPNVAAAVNGVAFCGTLAGQLFFGWLGDKLGRKKVYGITLIMMVLC |
| Motif 7 |  |
|  | TKTDAGYPPGIGVKNSLIMLGVINFLGMLFTFLVPEPKGKSLEELSGETE |
| Motif 8 |  |
|  | NFGLFSKEFMKRHGLHLLGTTSTWLLDIA |
| Motif 9 |  |
|  | FTVAFIDIMGRFAIQLMGFFFMTVFMFAL |
| Motif 10 |  |
|  | GGIVALIVSSIFDKKPSPAYSVDAAGST |
| Motif 11 |  |
|  | LILSKAGVFCAIVSHPADNLVSFLNNAKGATVGDAVKKIGL |
| Motif 12 |  |
|  | GKAGAIVGAFGLYAAQSQDK |
| Motif 13 |  |
|  | SVASGLSFGHKPKGV |
| Motif 14 |  |
|  | KVLQVEIEAEERAED |
| Motif 15 |  |
|  | PGFARGMSDGFPKFVKSEGFGGLYKGLAPLWGRQIPYTMMKFASFETIVE |
